# Impact of HER2 Status on Pathological Response after Neoadjuvant Chemotherapy in Early Triple-Negative Breast Cancer

**DOI:** 10.3390/cancers14102509

**Published:** 2022-05-19

**Authors:** Camille Domergue, Elodie Martin, Camille Lemarié, Pascal Jézéquel, Jean-Sebastien Frenel, Paule Augereau, Mario Campone, Anne Patsouris

**Affiliations:** 1 Medical Oncology Department, ICO Institut de Cancérologie de l’Ouest, 49000 Angers, France; paule.augereau@ico.unicancer.fr (P.A.); mario.campone@ico.unicancer.fr (M.C.); anne.patsouris@ico.unicancer.fr (A.P.); 2 Clinical Trial Sponsor Unit/Biometry, ICO Institut de Cancérologie de l’Ouest, 44800 Saint-Herblain, France; elodie.martin@ico.unicancer.fr; 3 Biopathology Department, ICO Institut de Cancérologie de l’Ouest, 44800 Saint-Herblain, France; camille.lemarie@ico.unicancer.fr; 4 Omics Data Science Unit, ICO Institut de Cancérologie de l’Ouest, 44800 Saint-Herblain, France; pascal.jezequel@ico.unicancer.fr; 5 Medical Oncology Department, ICO Institut de Cancérologie de l’Ouest, 44800 Saint-Herblain, France; jean-sebastien.frenel@ico.unicancer.fr

**Keywords:** triple-negative breast cancer, complete histologic response, HER2-low, prognosis

## Abstract

**Simple Summary:**

HER2-low (IHC 1+ or IHC 2+/ISH-) breast cancer (BC) is an emerging subtype of BC with promising results with antibody drug conjugate (ADC) in the metastatic setting. In the early setting, few data have been reported regarding the predictive and prognostic impact of HER2-low status in triple-negative BC (TNBC).

**Abstract:**

Purpose: Investigates the link between HER2 status and histological response after neoadjuvant chemotherapy in patients with early TNBC. Methods: We retrieved clinical and anatomopathological data retrospectively from 449 patients treated for the first time with standard neoadjuvant chemotherapy for early unilateral BC between 2005 and 2020. The primary endpoint was pathological complete response (pCR, i.e., ypT0 ypN0), according to HER2 status. Secondary endpoints included invasive disease-free survival (I-DFS) and overall survival (OS). Results: 437 patients were included, and 121 (27.7%) patients had HER2-low tumours. The pCR rate was not significantly different between the HER2-low group vs. the HER2-0 group (35.7% versus 41.8%, *p* = 0.284) in either univariate analysis or multivariate analysis adjusted for TNM classification and grade (odds ratio [OR] = 0.70, confidence interval [CI] 95% 0.45–1.08). With a median follow-up of 72.9 months, no significant survival differences were observed between patients with HER2-low tumours vs. patients with HER2-0 tumours in terms of I-DFS (*p* = 0.487) and OS (*p* = 0.329). Conclusions: In our cohort, HER2 status was not significantly associated with pCR in a manner consistent with data published recently on TNBC. However, the prognostic impact of HER2-low expression among TNBC patients warrants further evaluation.

## 1. Introduction

Triple-negative breast cancer (TNBC), defined by the lack of expression of oestrogen and progesterone receptors (ER and PR, respectively) and associated with the absence of HER2 overexpression/amplification, represents approximately 15% of cases of early stage invasive breast cancer [1]. Chemotherapy is the cornerstone of treatment in this phenotype, with no biomarkers that can identify the patients most likely to respond to cytotoxics. Targeted therapies have shown contrasting results, notably for anti-angiogenic [2] and anti-EGFR [3] therapies. More recently, PARP inhibitors in patients with a germline mutation of BRCA1/2 [4,5] and immunotherapy [6] have shown efficacy in early settings. TNBC is a very heterogeneous group of tumours, at the phenotypical, molecular, prognosis, and predictive levels. TNBC includes a large proportion of basal-like tumours identified by Perou and Sorlie and characterized by extensive proliferation, and poor prognosis with a risk of early relapse [7,8,9,10]. Lehmann et al. [11] initially described six molecular subtypes, subsequently refined into four subtypes including basal-like 1 and 2 (BL1, BL2), mesenchymal-like (M), and luminal androgen receptor (LAR) with differential responses to chemotherapy (Lehmann et al., 2016). Farmer et al. identified, by analysing 49 breast tumours, three subgroups: luminal, basal and molecular apocrine. The HER2 amplified tumours were distributed in the luminal and molecular apocrine subgroups [12], representing between 22 and 33% of TNBCs with strong expression of androgen receptors. Jézéquel et al. [13] distinguished three clusters by analysing the transcriptional profile of 107 cases of TNBC: C2 and C3 were distinguished by immune responses, and C1 was enriched in the luminal androgen receptor (LAR). In this last cluster (C1), molecular apocrine, correlation with PAM50 luminal B, and HER2-enriched subtypes were close, with high expression of the ERBB2 pathway. According to the 2018 ASCO and College of American Pathologists (CAP), HER2 protein overexpression assessed by immunohistochemistry (IHC) score 3+ or HER2 gene amplification, assessed by in situ hybridization (ISH) assay, is the predictive biomarker of HER2-targeted therapies in breast cancer, such as trastuzumab. A subpopulation of TNBC does not overexpress HER2 or show HER2 amplification but is molecularly enriched in genes of the “HER2” group, as previously seen. Recently, a new entity has emerged named HER2-low BC, defined by IHC 1+, or IHC 2+ without amplification assessed by ISH. It represents between 45 and 60% of HER2-negative BC tumours according to ASCO 4 CAP [14,15], which includes 37% of TNBC. The oncogenic role of HER2 in HER2-low BC is still unclear. Preclinical studies suggest that the activity of certain antibody drug conjugates (ADC) may be dependent on the expression levels of HER2 protein rather than on HER2 amplification [16], and some data seem to show promising results with trastuzumab deruxtecan in advanced HER2-low BC [17], rather than a bystander effect. These newer HER2 ADC have the potential to overcome HER2 expression heterogeneity [18]. In the neoadjuvant setting, pCR is associated with better relapse-free survival (RFS), especially in the TNBC phenotype [19,20,21]. Masuda et al. [22] reported the profile of response to anthracycline/taxane-based neoadjuvant chemotherapy (NACT) in 146 TNBCs, classified according to Lehmann’s subtypes, with the highest pCR rate presented by the BL1 subtype (52%) and the lowest in the BL2 and LAR subtypes (0% and 10%, respectively). Previous studies suggest that pCR rates are significantly higher for patients with HER2 overexpression, compared with patients with low HER2 expression [15], but there is not much data on the impact of HER2 low expression in response to chemotherapy, and what there is has rather contradictory results [23]. Finally, further studies have identified an elevated platelet to lymphocyte ratio (PLR), a marker of inflammation, as a very poor prognosis factor in terms of OS in TNBC (cut-off > 190) [24,25,26,27,28]. A high PLR is also associated with lower response to neoadjuvant chemotherapy [29,30,31]. The aim of the present study was thus to investigate the impact of HER2 status (HER2-low versus HER2-0) on the histological response after NACT in a cohort of patients with early TNBC. The secondary objectives were to evaluate the prognostic impact of HER2 status and baseline PLR in early TNBC.

## 2. Materials and Methods

### 2.1. Study Design

This retrospective study includes all patients treated with NACT for early TNBC between 2005 and 2020 at the ICO. Patients were included if they fulfilled the following criteria: (a) unilateral TNBC (defined by ER and PR < 10%, HER2 0 or 1+ in IHC or HER2++ in IHC with ISH negative); (b) T1-2, N0-3, M0 staging according to UICC criteria; (c) treated with NACT and surgery; (d) over the age of 18 years. Exclusion criteria were (a) metastatic or relapsed disease, (b) male patients, (c) not amenable to surgery, (d) radiotherapy performed before surgery, (e) previous or concomitant malignancies, (f) previously received adjuvant chemotherapy. The following information was recorded for each patient: (a) age at diagnosis; (b) clinical stage; (c) TNM stage and ultrasound tumour size; (d) histological type and axillary lymph node involvement; (e) Elston and Ellis grade; (f) hormone receptor status and HER2 status; (g) presence of tumour infiltrating lymphocytes (TILS); (h) mitotic account and Ki67; (i) type of surgery (conservative or mastectomy; sentinel node or axillary dissection); (j) biological data for calculating the PLR; (k) details of systemic neoadjuvant treatment; (l) adjuvant radiotherapy and (m) outcomes. pCR was defined as the absence of residual invasive cancer regarding breast and axillary lymph node after NACT, using Sataloff and the residual disease index known as the residual cancer burden (RCB) classification. The pCR was thus defined as classification RCB-0 and/or Sataloff’s TA-NA or TA-NB, i.e., ypT0 ypN0 [32]. We recorded data on androgen receptor or CK5/6 positivity, but this information was only available for 129 and 143 of the 449 patients, respectively, and was therefore not used in the data analysis.

### 2.2. Definition of the End Points

The main objective was to study the association between HER2 immunohistochemical status (HER2-low vs. HER2-0) and pCR after NACT in early TNBC. The primary endpoint was pCR, according to HER2 status. The secondary objectives were to compare pCR rates in HER2 1+ versus 2+ subgroups, compare early response rates, and estimate invasive disease-free survival (I-DFS), distant disease-free survival (D-DFS), OS, breast cancer specific survival and impact of the baseline PLR rate (high versus low with a cut-off equal to 190). Follow-up data were collected for each patient, including I-DFS (time from diagnosis to the earliest locoregional or distant disease recurrence, invasive controlateral cancer, second primary cancer, or death), D-DFS (defined as the time until metastasis or death), and OS (time from diagnosis to death from any cause) [33]. Patients who did not experience the event of interest were censored at their last follow-up.

### 2.3. Statistical Analysis

Categorical variables were described by the number and percentage of each modality of the variable. Continuous data were described by the median, minimum, and maximum. Comparisons between groups were made using Fisher’s exact test for categorical variables and the Kruskall–Wallis test for continuous variables. The Wilcoxon signed rank test was used to examine changes over time (pre- and post-chemotherapy). The analyses evaluating the associations between pCR and clinically relevant parameters were performed with univariate and multivariate logistic regression models; the ORs and corresponding confidence intervals (CIs) were reported. Survival data were estimated using the Kaplan–Meier method and presented with their 95% confidence intervals for the overall population and by group. Univariate analyses were performed using the log-rank test for categorical variables or the Cox proportional hazards model for continuous variables [34]. All analyses were performed using Stata^®^ version 16 and R software version 4.0.2. All tests used were two-sided with an α threshold at 5%. An independent ethics committee (CHU Angers) approved the study protocol (N° 2020-133).

## 3. Results

### 3.1. Patient and Tumour Characteristics 

**Figure 1:**
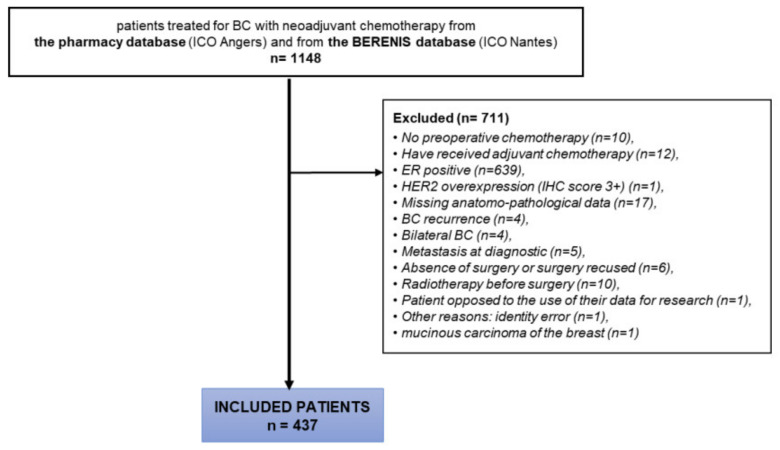
CONSORT diagram of the study.

A total of 437 out of 1148 patients were included into the study from 2005 to 2020 at ICO (Figure 1). Details of patient characteristics are found in Table 1. Of the 437 TNBC patients included, 121 (27.7%) had an HER2-low tumour (90 HER2 1+, 28 HER2 2+). Five patients were classified according to IHC status on postoperative histology in the absence of HER2 IHC status available on biopsies. The median age was 51 years (range: 42–62). Non-specific invasive carcinomas were predominant (95.2%). Most of the tumours (93.1%) were classified as ≥ T2 (5.8% T2; 24.5% T3; 12.8% T4) and 60.6% had node axillary involvement (49.1% N1, 10% N2, 1.4% N3). There was no significant association between HER2 status and stages T and N. mSBR grade was not significant between the two groups, with 73.3% of grade III in HER2-low vs. 77% in HER2-0 groups, while Ki67 was significantly higher in the HER2-low group with a mean of 60.9% (sd: 22.7) versus 49.0% (22.8) in the HER2- 0 group, however with missing data for 199 and 85 patients from these two groups, respectively (*p* = 0.008). Systemic NACT included a sequential of anthracyclin followed by taxanes in 95% of patients. The dose-dense regimen in this sequential was administered to 57 patients (13%), 9 patients in the HER2-low group (7.4%) and 48 patients in the HER2-0 group (15.2%). A total of 38 patients (8.7%) received associated platinum salts. The time from diagnosis to the beginning of chemotherapy and the number of postponements of treatment were well balanced in both groups.

### 3.2. Predictive Value of HER2 Status on pCR

The pCR rate was not significantly different between the HER2-low versus HER2-0 groups (35.5% versus 42.7%, *p* = 0.284) in univariate and multivariate analysis (Table 2). Only stages T3–T4 vs. T0 were significant in multivariate analysis. There was also no significant difference in pCR rate in the HER2 1+ and 2+ subgroups (Supplementary Table S1). The clinical and pathological characteristics of the patients after surgery are listed in Supplementary Table S2. Achieving a partial or complete response on early imaging during chemotherapy was not significantly associated with attaining a pCR (pCR 44.6% in HER-low vs. 50.0% in HER2-0, *p* = 0.522).

### 3.3. Prognostic Value of HER2 Status

**Figure 2:**
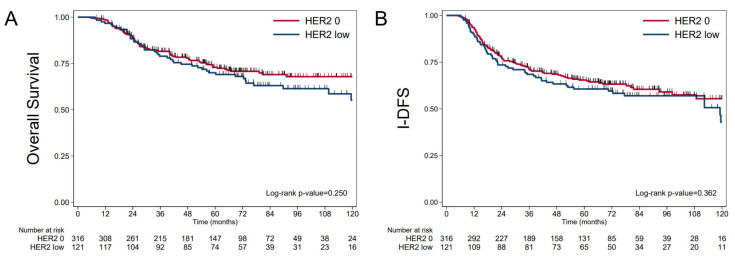
Outcomes data according to HER2 status (HER2-low versus HER-0). (**A**) OS, (**B**) I-DFS.

Median follow-up was 72.9 months (95% CI 69.9; 77.5) and median survival was not achieved. OS at 5 years was 72.02% (95%CI 67.22; 76.23). No association with OS was found (*p* = 0.329) between groups: 5-year OS was 70.00% (95%CI 67.22; 76.23) in the HER2-low group versus 72.9% (95%CI 67.1; 77.8) in the HER2-0 group (Figure 2). Five-year I-DFS was 63.99% (95%CI 59.08; 68.48) with median I-DFS at 124.3 months (95%CI 112.3; NR). The most frequent first event was metastatic relapse, which occurred in 115 patients (26.3%). No significant association with I-DFS was found between the two groups (*p* = 0.487): 5-year I-DFS was 60.6% (95%CI: 51.2; 68.8) in the HER2-low group, versus 65.4% (95%CI: 59.5; 70.6) in the HER2-0 group (Figure 2). The five-year D-DFS was 66.94% (95%CI: 62.07; 71.33), without reaching a significant difference between the two groups (*p* = 0.210): 63.1% in the HER2-low group vs. 68.5% in the HER2-0 group (Supplementary Table S3). Five-year specific survival was 73.19% (95%CI 68.44; 77.35) for the entire cohort.

### 3.4. Prognostic Value of PLR

A baseline high PLR rate (≥190) differed significantly depending on HER2 status: 37/113 (32.7%) and 60/284 (21.1%) in the HER2-low and HER2-0 groups, respectively (*p* = 0.02). At the end of chemotherapy, the rate of high PLR was considerably increased, with a rate of 75.5% (<0.001) in the entire cohort (Supplementary Table S4), 65.3% in the HER2-low, and 80.5% in the HER2-0 groups, with no significant difference between the two groupes (*p* = 0.376).

A baseline high PLR using a cut-off of 190 had lower OS in the HER2-0 versus HER2-low groups, without reaching significant difference, with 5-year OS rates of 58.7% and 74.7%, respectively, versus 76.5% and 67.1% in these same groups when associated with baseline PLR of less than 190 (Supplementary Table S5 and Figure S1). A baseline high PLR also had a lower 5-year I-DFS rate in the HER2-0 group compared to the HER2-low group with no statistical significance: 52.6% and 70.2%, respectively, versus 70.2% and 58.3% in these same groups when associated with baseline PLR of less than 190 (*p* = 0.010). Finally, baseline high PLR was associated with poor prognosis in terms of OS and I-DFS in the HER2-0 group, while the opposite trend was observed in the HER2 low group (Supplementary Figure S1).

## 4. Discussion

A total of 121 patients (27.7%) from our TNBC cohort were HER2-low. A lower rate of pCR was observed in the HER2-low versus HER2-0 groups, with no statistical significance (35.5% versus 42.7%, *p* = 192). No association with OS was found between groups (*p* = 0.329). Our results complete insights into the clinical characteristics of HER2-low BC. The proportion of HER2-low group patients was coherent with the literature data, according to Scott M. et al. at ASCO 2021, with 38% HER2-low patients in their cohort of 389 TNBC [35], and 36.5% (26.8% 1+ and 9.8% 2+) in the study by Schettini et al. [14]. In this study, basal-like tumours were mostly concentrated within the IHC 0 (43.7%) and TNBC (84.7%) groups compared to IHC 2+ (9.8%), IHC 1+ (15.2%), HER2-low (13.4%) and HR-positive tumours (3.9%). Braso Maristany et al. observed no significant difference regarding subtypes identified by PAM50 distribution between HER2-0 and HER2-low in a cohort of 80 patients (*p* = 0.091) treated for BC (28.8% TNBC and 54.4% HR+) [36]. HER2- low tumours were more frequently found within HR-positive disease compared to TNBC (65.4% vs. 36.5%, *p* < 0.001, also reflecting the proportion of these phenotypes in BC [14]. Lehmann’s classification identified most TNBC as basal-like (80.6%). Interestingly, the LAR subtype was predominantly identified as either HER2-enriched (74%) or luminal B (14%) [13,37,38].

The pCR rate in the present cohort of TNBC was 42.7% of the 437 TNBC, which is not so different from the pCR observed in the GeparTrio study, of 509 cases of TNBC treated in the neoadjuvant setting with anthracyclines and taxanes, with a pCR rate of 39% [39,40]. In our cohort, the pCR rate was not significantly different between the HER2-low versus HER2-0 groups (35.5 % versus 42.7 %, respectively, *p* = 0.284) in univariate analysis, and even in multivariate analysis adjusted for grade, only stage T3-T4 was significantly associated with a poor response. There are few data in the literature regarding the histological response after NACT of HER2-low and HER2-0 tumours. In a study published in 2012 [41], Wang et al. showed that the pCR rate in a cohort of HER2-low patients treated with anthracycline-based NACT was 9.6% (out of 229 patients). Santonja et al. looked at the correlation between Lehmann’s classification and the achievement of pCR in neoadjuvant therapy in a cohort of 125 TNBCs (non-basal-like included 5 HER2-enriched and 1 luminal A). Despite small patient numbers, it was shown that LAR patients presented a lower rate of pCR (14.3% pCR in LAR versus 41.9% in other subtypes combined, *p* = 0.07) [42]. In another retrospective study of 146 TNBC patients who received a sequential of anthracycline and taxanes, the pCR rate was 28%, with significant differences between subtypes: the pCR rate in the BL1 subtype was the highest (52%), while those in the BL2, LAR, and MSL subtypes were 0%, 10%, and 23%, respectively [18]. Similarly, Echavarria et al. observed that among patients pretreated with NACT, the BL1 subtype had the highest pCR rate (65.6%), followed by BL2 (47.4%), while the LAR subtype had a significantly lower pCR rate (21.4%) [43]. Recently, a pooled analysis of 2310 patients with HER2 non-amplified early BC from four prospective neoadjuvant clinical trials, published by Denkert *et* *al.*, included 1162 patients with TNBC (395 patients HER2-low and 767 HER2-0). They observed no difference in terms of pCR rate in the TNBC subgroup: 45.6 % in the HER2-low group vs 44.9 % in the HER-0 group (*p* = 0.51), while pCR was significantly lower in the HER2-low patients (17.5% versus 23.6%; *p* = 0.024) in the hormone-receptor-positive subgroup [44]. Similarly, Moura Leite et al. recently reported a cohort of 855 HER2 non-amplified patients with 313 cases of TNBC treated with NACT, included 49 HER2-low, with no difference in terms of pCR rate in relation to HER2 status: 51% versus 47% in HER2-low versus HER2-0, respectively (*p* = 0.64) [45]. To note, in this last cohort, almost half of the patients had received a dose dense regimen, and half of the patients had also received carboplatin in both the HER2-low and HER2-0 groups. In our cohort, in HER2-0 and HER2-low, only 15.2% and 7.4% received a dose dense regimen and 10.4% and 4.1% received platinum salts. At the last San Antonio symposium, Reinert et al. reported in a cohort of 122 TNBC patients, a higher, although non-statistically significant, pCR rate in HER2-0 versus HER2-low tumours (56% vs. 39%, *p* = 0.09) [46].

In our cohort, with median follow-up of 72.9 months, a trend with lower outcomes were observed in the HER2-low group but with no significant association with I-DFS or OS found between the HER2-low and HER2-0 groups. Rossi et al. studied the prognostic differences between HER2 0/1+/2+ tumours [47] in a cohort that included 15% of TNBC, and observed at diagnosis larger tumours, frequently more proliferative tumours (higher-grade, higher Ki-67 rate), and more extensive axillary lymph node involvement in patients with tumours with HER2 scores of 0 and 1+, compared to HER2 score 2, regardless of HR (hormone receptor) status. The 5-year DFS rates were 86%, 84%, 62% and 63% for patients with tumours categorized as HER2 0, 1+, 2+; and HER2 amplified (2+, ISH+ or 3+), respectively. HER2-low status was associated in the cohort published by Denkert et al. with better outcomes with a 3-year DFS of 84.5% and a 3-year OS of 90.2% vs 74.4% and 84.3% in the HER2-0 group respectively, with median follow-up of 46.6 months (*p* = 0.0076 and *p* = 0.016) [44]. In contrast, Moura Leite et al. observed no significant prognostic value of HER2-low status, with 5-year RFS rates of 75.6% versus 70.8% (*p* = 0.23) for TNBC with HER2-low versus HER2-0, and 5-year OS of 79.1% versus 80.3% (*p* = 0.71), respectively after a median of 59 months [45]. Surprisingly, the HER2-low group in our cohort showed outcomes slightly lower than in the HER2-0 group, without reaching a significant difference between the two groups. Only higher proliferation, known to be associated with relapse, in the HER2-low group, especially regarding the Ki67 rate, differed from the HER2-0 group (median 64% vs 50% in the HER2-0 group, *p* = 0.008). Ki67 was lower on average in the Rossi et al. cohort [47] at 20% for HER-0 and 1+ and 26% for 2+, but more than 80% of these patients were RH+ in this study. At the molecular level, Shettini et al. reported a downregulation of proliferation-related genes in the HER2-low group. Ki-67 has been assayed in many studies as a predictive marker of response in early BC, however conflicting results have been published [48,49,50,51]. Some authors have investigated whether its level correlates with the achievement of pCR: in one study, the three groups of Ki67 ≤15% versus 15.1%-35% versus >35% had pCR-rates of 10%, 22.4%, and 39.0% in TNBC, respectively [51]. Nevertheless, in the study by Moura Leite et al., median Ki67 was 70% in HER2-low groups vs. 60% in HER2-0 (*p* = 0.80), and the proportion of grade III tumours did not differ between the two groups (67.3% in HER2-low vs. 67.8%, *p* = 0.91). Too much data was missing on proliferation markers (65%) to make a hypothesis in our cohort. Regarding other biological parameters, a high PLR rate at baseline seems to have a prognostic value. It has been previously shown that tumour cells induce the synthesis of the platelet stimulating factors that promote tumour growth [52] through systemic inflammation. Thisis associated with the release of several pro-inflammatory mediators such as interleukin, known to stimulate megakaryocyte proliferation leading to thrombocytosis [53]. Chemotherapy influences these parameters through its bone marrow toxicity. Similarly, the number of circulating lymphocytes reflects the systemic inflammation, tumour suppressive activity, and immunomodulation induced by chemotherapy. In light of this, patients with low PLR might have a better prognosis, thanks to better antitumour activity. A high PLR (≥190) at baseline in our cohort was associated with lower OS without statistical significance in the HER2-0 group, while the opposite trend was observed in the HER2-low group, with no difference in the PLR rate at the end of treatment. It would be interesting to conduct further studies on these subjects to see if there is an association between evolution in PLR levels during chemotherapy and survival, reflecting the immunomodulation induced by the treatments. Surprisingly, rates of high and low PLR increased with the same ratio in the HER2-low and HER2-0 groups. Kim et al. studied dynamic changes in PLR ratio and observed that a low PLR value at pre- and post-systemic treatment was significantly associated with better prognosis [54]. Further research is needed to determine the timing of evaluation of the PLR ratio and its prognostic value. TNBC with HER2-low status is probably a heterogeneous entity; HER2 expression can in fact present a variable profile, with considerable intra-tumoral heterogeneity [55,56]. This heterogeneity has been explored in TNBC by single cell analysis and may be associated with a decreased likelihood of achieving a pCR [57,58]. Trastuzumab deruxtecan seems to show better efficacy in score 2+ than score 1+ [17]. Current clinical trials involving HER2-ADCs suggest promising results in the treatment of HER2-low expressing BC, especially for ADCs responsible for the by-stander effect, with more efficacy in case of intra-tumoral heterogeneity. The study included a large cohort of patients with TNBC, bi-centrically. Some limitations need to be notified, including the retrospective nature of this study with outcomes affected by differences in terms of patient characteristics. On the other hand, 2/3 of the patients were HER2-0, which does not represent the characteristics of the HER2 negative population but is consistent regarding the TNBC phenotype. Finally, a major strength of the present work is that it is reported with significant hindsight regarding OS, with median of follow-up of 72.9 months.

## 5. Conclusions

To our knowledge, our study is the third recent cohort with response data in HER2-low TNBC after NACT. In our cohort, HER2-low status had no significant prognostic value on survival and no predictive effect on pCR after NACT. In addition, the trend towards poorer survival contrasts with published data in the HER2-low group, although without reaching significance and with significant follow-up. The prognostic value of HER2-low expression warrants further evaluation. We hypothesize that HER2-low TNBC is a heterogeneous entity with the interest of studying the dynamic changes on the residual disease in this entity after chemotherapy, and this needs to be characterized at the molecular level.

## Figures and Tables

**Table 1:** Baseline patient characteristics.

	HER2-0 (*n* = 316)	HER2-Low (*n* = 121)	*p-Value*	Total (*n* = 437)
**Age at diagnostic**				
Median (range)	51.0 (25–89)	52.0 (22–89)		51.0 (22–89)
**Histological type**				
Non-specific	304 (96.2%)	112 (92.6%)		416 (95.2%)
Invasive lobular	4 (1.3%)	3 (2.5%)		7 (1.6%)
Apocrine	1 (0.3%)	2 (1.7%)		3 (0.7%)
Other histological type	7 (2.2%)	4 (3.2%)		11 (2.5%)
**Stage T**			*p = 0.053*	
T0-T1	15 (4.7%)	15 (12.4%)		30 (6.7%)
T2	178 (56.3%)	66 (54.5%)		252 (56.1%)
T3	81 (25.6%)	26 (21.5%)		110 (24.5%)
T4	42 (13.3%)	14 (11.6%)		57 (12.7%)
**Stage N**			*p = 0.662*	
N0	122/315 (38.7%)	50/121 (41.3%)		172 (39.4%)
N+	193/315 (61.3%)	71/121 (58.7%)		264 (60.6%)
**RE**				
0	295/315 (93.7%)	116/120 (96.7%)		423/447 (94.5%)
1–10%	20/315 (6.3%)	4/120 (3.3%)		24/447 (5.5%)
**HER2 status**				
1+	0	90/119 (76.3%)		
2+	0	28/119 (23.7%)		
**mSBR grade**			*p = 0.452*	
Grade II	72/313 (23%)	32/120 (26.7%)		104/433 (24%)
Grade III	241/313 (77%)	88/120 (73.3%)		329/433 (76%)
**Mitotic index (/mm^2^)**				
Median (range)	8.1 (1.1–31.5)	8.8 (0.0, 30.0)		8.3 (0.0, 31.5)
Missing	254	99		353
**Ki67 (%)**			***p = 0.011***	
Median (range)	50.0 (10.0, 90.0)	64.0 (10.0, 95.0)		52.0 (10.0, 95.0)
Missing	199	85		284
**Neoadjuvant chemotherapy**			*p = 0.013*	
Anthracycline-taxane (A-T)	301/316 (95,3%)	114/121 (94,2%)		415/437 (95%)
A-T dose dense regimen	48/316 (15.2%)	9/121 (7.4%)		57/437 (13%)
Platinum salts	33/316 (10.4%)	5/121(4.1%)		38/448 (8.7%)
**Mammary surgery**			*p = 0.226*	
Mastectomy	107 (33.1%)	50 (39.7%)		157 (35.0%)
Conservative	216 (66.9%)	76 (60.3%)		292 (65.0%)
**Germline mutation**				
Number of screened patients	125	48		173
BRCA1	18 (14.4%)	8 (16.7%)		26 (15.0%)
BRCA2	9 (7.2%)	1 (2.1%)		10 (5.8%)
PALB2	1 (0.8%)	0 (0.0%)		1 (0.6%)
No identified mutation	97 (77.6%)	39 (81.3%)		136 (78.6%)

**Table 2:** pCR according to HER2 status (HER2-low versus HER2-0); univariate and multivariate analysis according to HER2 status.

	HER2-0		HER2-Low		Total	
	(*n* = 316)		(*n* = 121)		(*n* = 449)	
**pCR (i.e., ypT0 ypN0)**				*p = 0.192*		
No	181 (57.3%)		78 (64.5%)		259 (59.3%)	
Yes	135 (42.7%)		43 (35.5%)		178 (40.7%)	
	**Univariate**			**Multivariate** (*n* = 432)		
	OR	IC 95%	*p*-value	OR	IC 95%	*p-value*
**HER2**						
HER2 0	1.00			1.00		
HER 1+ or 2+	0.74	[0.48;1.14]	*0.172*	0.66	[0.42; 1.03]	***0.066***
**Stade N**						
N0	1.00			1.00		
N+	1.01	[0.68; 1.49]	*0.965*	1.08	[0.42; 1.03]	*0.700*
**Stade T**						
T0-T1	1.00			1.00		
T2	0.61	[0.28; 1.31]	*0.201*	0.60	[0.27; 1.32]	*0.207*
T3-T4	0.37	[0.17; 0.81]	***0.014***	0.35	[0.16; 0.80]	***0.012***
**mSBR grade (biopsy)**						
Grade II	1.00			1.00		
Grade III	1.08	[0.69; 1.70]	*0.729*	1.07	[0.72; 1.78]	*0.773*

## Data Availability

The datasets used and/or analysed during the current study are available from the corresponding author on reasonable request. The datasets supporting the conclusions of this article are included within the article (and its additional files).

## Supplementary Materials

The following are available online at https://www.mdpi.com/article/10.3390/cancers14102509/s1, Table S1. Association between HER2-low (HER2 1+ versus HER2 1+) and pCR, Table S2. Clinicopathological characteristics of the tumours after surgery, Table S3. D-DFS according to HER2 status, Table S4. Evolution of PLR and NLR at baseline and after treatment, Table S5. Outcomes according to baseline PLR et HER2 status, Figure S1. Outcomes according to baseline PLR. (A) OS and (B) I-DFS.
