# Supplementary material for: Impact of HER2 Status on Pathological Response after Neoadjuvant Chemotherapy in Early Triple-Negative Breast Cancer
Source: Cancers (Basel). 2022 May 19;14(10):2509. doi: 10.3390/cancers14102509 (PMC9139240; doi:10.3390/cancers14102509)
Supplement: Supplementary file 1 [file cancers-14-02509-s001.zip › cancers-1675511-supplementary.pdf]

# SUPPLEMENTARY DATA

**Supplementary Table S1.** Association between HER2-low (HER2 1+ versus HER2 1+) and pCR

**Supplementary Table S2.** Clinicopathological characteristics of the tumours after surgery

**Supplementary Table S3.** D-DFS according to HER2 status

**Supplementary Table S4.** Evolution of PLR and NLR at baseline and after treatment

**Supplementary Table S5.** Outcomes according to baseline PLR et HER2 status

**Supplementary Figure S1.** Outcomes according to baseline PLR. A) OS and B) I-DFS

**Supplementary Table S1. Association between HER2-low (HER2 1+ versus HER2 1+) and pCR.**

| | HER2 1+ (N = 95) | HER 2+ (N = 31) | p-value |
|---|---|---|---|
| **pCR** | | | *0.673* |
| No | 60 (63.2%) | 21 (67.7%) | |
| Yes | 35 (36.8%) | 10 (32.3%) | |

**Supplementary Table S2. Clinicopathological characteristics after surgery.**

| | **HER2-0** (N = 323) | **HER2-low** (N = 126) | **Total** (N = 449) |
|---|---|---|---|
| **mSBR grade** | | | |
| Grade II | 5/38 (13.2%) | 1/10 (10.0%) | 6/48 (12.5%) |
| Grade III | 33/38 (86.8%) | 9/10 (90.0%) | 42/48 (87.5%) |
| **RE** | | | |
| 0 | 91/97 (93.8%) | 36/39 (92.3%) | 127/136 (93.4%) |
| 1-10% | 6/97 (6.2%) | 3/39 (7.7%) | 9/136 (6.6%) |
| **IHC HER2** | | | |
| 0 | 83/88 (94.3%) | 18/38 (47.4%) | 101/126 (80.2%) |
| 1+ | 4/88 (4.5%) | 15/38 (39.5%) | 19/126 (15.1%) |
| 2+ | 1/88 (1.1%) | 5/38 (13.2%) | 6/126 (4.8%) |
| **Ki67 (%)** | | | |
| Median (Q1, Q3) | 31.0 (8.0, 50.0) | 52.0 (30.0, 70.0) | 34.0 (10.0, 60.0) |
| **TILs** | | | |
| Yes | 103/315 (32.7%) | 37/124 (29.8%) | 140/439 (31.9%) |
| **Interval response imaging** | | | |
| Stable disease | 87/213 (40.8%) | 28/88 (31.8%) | 115/301 (38.2%) |
| Progress | 5/213 (2.3%) | 3/88 (3.4%) | 8/301 (2.7%) |
| Partial or complete response | 121/213 (56.8%) | 57/88 (64.8%) | 178/301 (59.1%) |

**Supplementary Table S3.** D-DFS according to HER2 status.

| | events/n | 5 years D-DFS rate [95%CI] |
|---|---|---|
| **HER2** | | |
| HER2 0 | 97/316 | 68.5% [62.7;73.6] |
| HER2 1+ ou 2+ | 49/121 | 63.1% [53.7;71.1] |

**Supplementary Table S4. Evolution of PLR at baseline and after treatment.**

| | Median difference (after – before CT) | Ratio (after/ before CT) | Evolution rate % | P value (paired test*) |
|---|---|---|---|---|
| **PLR** | | | | |
| Median (range) | 110.3 (-274.5; 1412.4) | 1.8 (0.3; 9.3) | 75.5 (-71.7, 826.4) | **<0.001** |
| Missing | 51 | 51 | 51 | |

*Wilcoxon signed Rank test; *CT: chemotherapy*

**Supplementary Table S5. Outcomes according to baseline PLR and HER2 status.**

| | | **OS** | | | |
|---|---|---|---|---|---|
| | n | HR [CI 95%] | p | HR [CI 95%] | p |
| **Baseline PLR** | | | | | |
| **--- ≥ 190** | | | | | |
| HER2-0 | 60 | 1.00 (ref) | | 2.28 [1.40;3.71] | 0.001 |
| HER2-low | 37 | 0.54 [0.27;1.09] | 0.085 | 1.24 [0.66;2.33] | 0.508 |
| **— < 190** | | | | | |
| HER2-0 | 224 | 0.44 [0.27;0.71] | 0.001 | 1.00 (ref) | |
| HER2-low | 76 | 0.73 [0.42;1.25] | 0.246 | 1.65 [1.05;2.62] | 0.031 |

| | | **i-DFS** | | | |
|---|---|---|---|---|---|
| | n | HR [CI 95%] | p | HR [CI 95%] | p |
| **Baseline PLR** | | | | | |
| **--- ≥ 190** | | | | | |
| HER2-0 | 60 | 1.00 (ref) | | 2.03 [1.31;3.16] | 0.002 |
| HER2-low | 37 | 0.58 [0.31;1.10] | 0.094 | 1.18 [0.68;2.08] | 0.554 |
| **— < 190** | | | | | |
| HER2-0 | 224 | 0.49 [0.32;0.77] | 0.002 | 1.00 (ref) | |
| HER2-low | 76 | 0.74 [0.45;1.23] | 0.249 | 1.51[1.00;2.29] | 0.050 |

**Supplementary Figure S1. Outcomes according to baseline PLR. (A) OS and (B) I-DFS.**

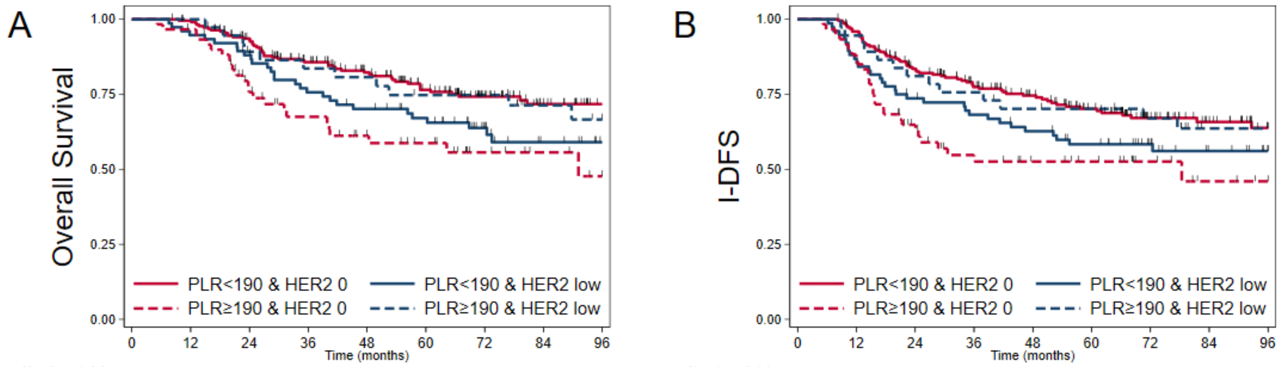


| Number at risk | 0 | 12 | 24 | 36 | 48 | 60 | 72 | 84 | 96 |
|---|---|---|---|---|---|---|---|---|---|
| PLR<190 & HER2 0 | 224 | 220 | 194 | 161 | 137 | 111 | 74 | 53 | 34 |
| PLR<190 & HER2 low | 76 | 72 | 65 | 55 | 50 | 44 | 28 | 20 | 14 |
| PLR ≥190 & HER2 0 | 60 | 57 | 40 | 32 | 25 | 21 | 12 | 8 | 5 |
| PLR ≥190 & HER2 low | 37 | 37 | 32 | 30 | 28 | 24 | 23 | 15 | 13 |

| Number at risk | 0 | 12 | 24 | 36 | 48 | 60 | 72 | 84 | 96 |
|---|---|---|---|---|---|---|---|---|---|
| PLR<190 & HER2 0 | 224 | 212 | 173 | 146 | 123 | 101 | 65 | 46 | 29 |
| PLR<190 & HER2 low | 76 | 66 | 55 | 50 | 45 | 39 | 26 | 19 | 13 |
| PLR ≥190 & HER2 0 | 60 | 52 | 34 | 25 | 20 | 17 | 10 | 5 | 3 |
| PLR ≥190 & HER2 low | 37 | 35 | 30 | 28 | 25 | 23 | 21 | 13 | 12 |
